# Intestinal parasites among food handlers of food service establishments in Ethiopia: a systematic review and meta-analysis

**DOI:** 10.1186/s12889-020-8167-1

**Published:** 2020-01-16

**Authors:** Yonas Yimam, Ambachew Woreta, Mehdi Mohebali

**Affiliations:** 1 Department of Medical Parasitology and Mycology, School of Public Health, Tehran University of Medical Sciences, Tehran, Iran; 2 Department of Biology, Faculty of Natural and Computational Sciences, Woldia University, Woldia, Ethiopia; 3 Department of Microbial Cellular and Molecular Biology, College of Natural Sciences, Addis Ababa University, Addis Ababa, Ethiopia; 4 Research Center for Endemic Parasites, Tehran University of Medical Sciences, Tehran, Iran

**Keywords:** Food handlers, Intestinal parasites, Ethiopia, Systematic review, Meta-analysis

## Abstract

**Background:**

Intestinal parasites remain considerable public health problems in low-income countries where poor food hygiene practice is common. Food handlers, people involved in preparing and serving food, working with poor personal hygiene could pose a potential threat of spreading intestinal parasites to the public in a community. The aim of this systematic review and meta-analysis was, therefore, to synthesize the pooled prevalence estimate of intestinal parasites and associated pooled odds ratio of hygienic predictors among food handlers of food service establishments in Ethiopia that could aid to further bringing down the burden of intestinal parasites and it can also be used as a springboard for future studies.

**Methods:**

We searched exhaustively for studies Published before 20 April 2019 using eight Databases; PubMed, Science Direct, Web of Science, Scopus, Embase, Google Scholar, ProQuest, and Ovid MEDLINE® complemented by the gray literature search. In the final synthesis, we included twenty study reports. We used the Cochrane Q test and I^2^ test to assess heterogeneity of studies, while we employed a funnel plot followed by Egger’s regression asymmetry test and Begg rank correlation methods to evaluate publication bias. We also performed a point estimates and 95% confidence interval for each study using STATA version 14 statistical software.

**Results:**

The overall pooled prevalence estimate of intestinal parasites among food handlers of food service establishments in Ethiopia was 33.6% (95%CI: 27.6–39.6%). Among ten intestinal parasites identified from food handlers, *Entamoeba histolytica/ dispar* (11, 95%CI: 7.9–14.1%) and *Ascaris lumbricoides* (8.8, 95%CI: 6.4–11.2%) were the most predominant intestinal parasites. Food handlers who washed hands after toilet use had 54% (OR, 0.46, 95% CI: 0.23–0.94) protection from intestinal parasites compared to those who did not.

**Conclusions:**

This study revealed that intestinal parasitic infections are notable among food handlers of food service establishments in Ethiopia, which may be a risk for transmitting intestinal parasites to food and drinks consumers through the food chain. Thus, periodic stool checkup, training on intestinal parasitic infections and personal hygiene should be applied to reduce public health and socio-economic impacts of parasitic infections.

## Background

Gastrointestinal parasitic infections have a widespread distribution across the globe with the highest-burden in developing countries where poor personal hygiene, environmental sanitation, socio-economic, demographic, and health-related behaviors have documented to influence their transmission [1]. The most familiar way of the spread of intestinal parasitic infections is through ingestion of contaminated food and water, yet they may also spread from human to human via fecal-oral contact [2]. Globally, intestinal parasites infect approximately one-third of the total world population, with the highest burden in tropics and subtropics [3]. In the world, an estimated 1.2 billion, 795 million, 740 million, 500 million, and 2.8 million people are infected with *Ascaris lumbricoides*, *Trichuris trichiura*, hookworm [4], *Entamoeba histolytica* and *Giardia lamblia* [5], respectively.

In Ethiopia, the burden of intestinal parasites is substantially high. About a third of (26million), one quarter (21 million), one in eight (11 million) Ethiopian people harbour *Ascaris lumbricoides*, *Trichuris trichiura,* and hookworm, respectively. Consequently, Ethiopia bears the second, the third, and fourth highest burden of ascariasis, hookworm, and trichuriasis, respectively, in sub-Saharan Africa [6].

Food handlers, individuals engaged in preparing and serving foods, infected with gastrointestinal parasitic infections, and practicing poor personal hygiene could be dangerous sources of transmission to the society for intestinal parasites. Because food handlers infected with intestinal parasites show sub-clinical signs and are asymptomatic carriers, they are unaware of their potential role in the spread of infections, and subsequently, it hinders control and elimination [2]. Moreover, the impact of food handlers on spread of intestinal parasites is high since they can directly or indirectly transmit infections via food, water, nails, and fingers to a large number of food and drink consumers of food service establishments like restaurants, hotels, factories, canteens, schools, hospitals, prisons, or other places where food prepared and served to many people [7, 8].

Apart from socio-economic factors, other factors like availability of clean water, the survival of the environmental stages of the parasites, personal and public hygiene practices play a central role in the transmission of intestinal parasites [9, 10]. Ethiopia has one of the bottommost clean water supply and latrine coverage [11]. Studies carried out in Ethiopia indicated that personal hygienic factors like hand washing after toilet use, medical cheek up including stool examinations, and knowledge about intestinal parasites contribute to the prevalence of intestinal parasite infections among food handlers of food service establishments [12–14].

In Ethiopia, fragmented and dispersed studies conducted on the prevalence of intestinal parasitic infections among food handlers of food service establishments. The result of these studies showed variation and inconsistency in the prevalence of intestinal parasitic infections among food handlers: 10.9 to 45.3% in Ethiopian university cafeterias [15–22], 61.9% in prisons [14], 35% in orphanage centers [23], (32.3%) public hospitals [24], 14.5 to 44% in restaurants and cafeterias [13, 25–28]. According to the result of those studies, the prevalence of intestinal parasitic infections among food handlers of food service establishments was wide-ranging. However, the possible reasons for heterogeneity and inconsistency in the prevalence of intestinal parasites among food handlers of food service establishments and associated hygienic predictors have not yet been explored in Ethiopia. Thus, the main objective of this first of its kind systematic review and meta-analysis was to estimate the pooled prevalence of intestinal parasites and associated pooled odds ratio of hygienic predictors among food handlers of food service establishments in Ethiopia.

## Methods

This Systematic Reviews and Meta-analyses was performed following the PRISMA guideline (Preferred Reporting Items for Systematic Reviews and Meta-Analyses) [29]. We used the PRISMA guideline for the inclusion of potentially related studies to the outcome of interest. The outcome of interest was the prevalence of intestinal parasitic infections among food handlers of food service establishments in Ethiopia and their hygienic predictors. Then we extracted data from relevant studies and meta-analyzed to provide pooled prevalence estimates of intestinal parasites and associated pooled odds ratio of hygienic predictors among food handlers of food service establishments in Ethiopia.

### Search strategy

We performed a comprehensive search of databases to identify relevant studies published in PubMed, Science Direct, Web of Science, Scopus, Embase, Google Scholar, ProQuest Dissertations & Theses, and Ovid MEDLINE® using Medical Subject Heading (MeSH) terms and keywords. The search terms include “intestinal parasites,” “Parasitic Intestinal Disease (MeSH),” “Food handlers,” and “Ethiopia.” For example, we searched in all field of PubMed using; (((intestinal parasites OR Parasitic Intestinal Diseases)) AND Food handlers) AND Ethiopia (Additional file 1: Table S1).

Moreover, we screened reference lists of all selected studies for studies related to intestinal parasite infections among food handlers in Ethiopia and we retrieved gray literature search of unpublished M.Sc. Thesis and Ph.D. dissertations of Ethiopian Universities using Google and Google Scholar. A search of articles was performed on April 20, 2019, using the English language for clarity, understandability, and simplicity of interpretations. We imported and stored all searched articles in EndNote X7 software (Thompson Reuter, CA, USA) for management. After removing duplicated articles, we did the screening of pertinent studies in two rounds. In the first round, we read titles and abstracts, and we excluded articles that were not suitable for the outcome of interest. In the second round, we screened full text of articles that were eligible for the first round by strictly applying inclusion/exclusion criteria. Lastly, we approved full-text studies for the final synthesis. Two authors were involved in various steps of literature search, screen, and selection of eligible studies. Any inconsistency in search and selection of studies was resolved by agreement.

### Inclusion and exclusion criteria

In this systematic review, we included studies conducted in Ethiopia and reported the prevalence of intestinal parasites and associated hygienic factors in food handlers of food service establishments irrespective of study designs, time and geographical regions of studies and publication condition (both published and unpublished studies). We included Literature published in the English languge. In this systematic review and meta-analysis, the outcome of interest (raw data) was sample size and number of individuals infected with intestinal parasites which help to estimate the pooled prevalence of intestinal parasites in food handlers of food service establishments. The second outcome of interest was hygienic determinates of intestinal parasitic infections among food handlers that help to calculate the pooled odds ratio. We excluded studies if they were reviews; sample size less than 35 and if they did not contain the outcome of interest.

### Data extraction and quality assessment

For data extraction of each study, we used a pre-designed data extraction excel sheet, and we extracted the following data: author/s, publication year, study area, study setting, total sample size, the overall number of positive, the type of specific intestinal parasites and hygienic practices. We did quality assessments of the studies using Hoy 2012 ten criteria that address internal and external validity [30]. The tool contained 4 external validity items such as I) representation, II) sampling frame, III) random selection, and, IV) non response bias and six internal validity assessment items were: I) data collection, II) appropriateness of case definition, III) reliability and validity of study instrument, IV) method of data collection, V) duration of prevalence period, and VI) correctness of numerator and denominator. We rated each item as the low, moderate or high risk of bias. Uncertain was considered as high risk of bias. Summary of risk of bias assessment was then rated based on the number of the high risk of bias per study; Low (≤ 2), moderate (3–4), and high (≥ 5) (Additional file 2).

### Data analysis

For the meta-analysis of proportion, the inverse variance method is widely used and works for prevalence proportions around 0.5. However, two problems occur when the proportions get closer to the limits of the zero and one range: I) confidence interval does not preclude confidence limits outside the 0–1 range. II) a study gets a large weighting when the proportion becomes too small or too big [31]. Therefore, we transformed point estimates of studies by variance stabilizing double arcsine transformation using the following formula: t = arcsin (sqrt r/(n + 1))) + arcsin(sqrt ((r + 1)/(n + 1))), where t = transformed prevalence, r = positive numbers, and n = sample size; se(t) = sqrt(1/(n + 0.5)), where se = standard error and the back transformation to a proportion is done using: p = (sin(t/2))^2^. We used STATA 14 statistical software for statistical and meta-analysis. We calculated prevalence for each study with 95% confidence intervals (CI) followed by pooled prevalence estimate and associated pooled odds ratio. In addition to a forest plot, we evaluated heterogeneity among studies using Q statistic and I^2^ index, assuming that I^2^ values of 25, 50, and 75% represented as low, medium, and high heterogeneity, respectively [32]. Also, we performed meta-regression by considering the year of publication and sample size to detect the potential source of heterogeneity. Sub-group analyses were also carried out based on specific species of intestinal parasites and geographical regions where studies were conducted. For the detection of publication bias, we used direct observation of funnel plot symmetry and Egger’s regression asymmetry test [33] and Begg rank correlation methods [34], respectively. We carried out a sensitivity analysis to assess the robustness of the results.

## Results

**Fig. 1:**
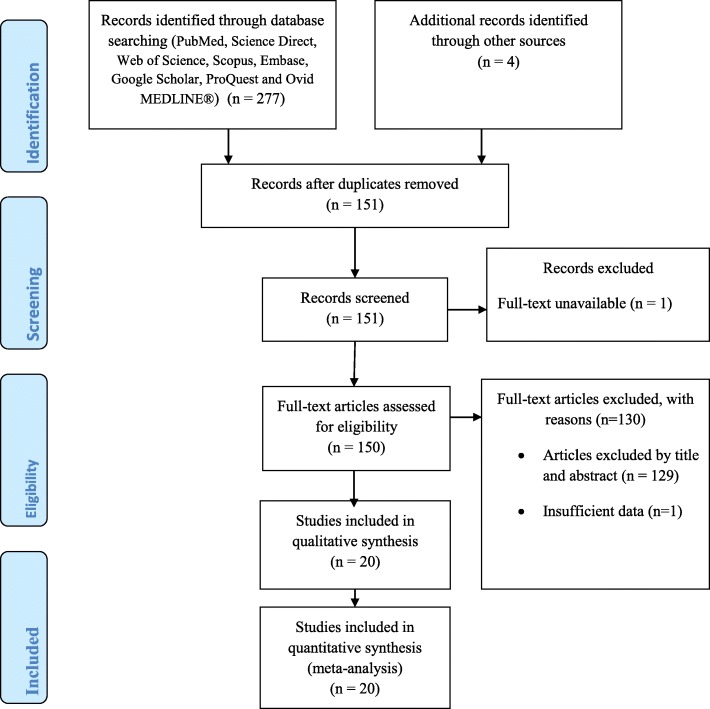
PRISMA flowchart for the selection of eligible studies on intestinal parasitic infections among food handlers at food service establishments in Ethiopia, 2019

In total, 281 published and unpublished study reports identified. Out of 281 articles, 130 were found to be duplicates. After removing 130 duplicates, 151 articles screened for titles and abstract screening. One study was removed due to the unavailability of the full text. Out of 150 full-text studies, 129 studies were excluded based on title and abstract screening, and 1 study was excluded due to the insufficiency of its data. Finally, 20 studies fulfilling inclusion criteria were included for the final analysis (Fig. 1).

Table 1 presents 20 studies that were eligible and included in this Systematic Review and Meta-analysis. Studies included for this meta-analysis were published from 2001 to 2019. Out of 20 studies, two studies [23, 24] were unpublished, and 18 studies were published. Prevalence of intestinal parasitic infections (were ranged from 13%(95%CI: 10–16%) to 58%(49–68%). For estimations of pooled prevalence of intestinal parasitic infections, a total of 5234 food handlers were involved. Concerning the study settings, twelve studies were from the public university cafeterias, five from town’s restaurants and cafeterias, one from prison cafeterias, one from public hospitals cafeterias and one study from the orphanage centers cafeterias. Regarding the risk of bias assessments, sixteen studies (80%), three studies (15%) and one study (5%) had a low risk of bias assessment, medium risk of bias and high risk of bias, respectively.

**Table 1:** List and characteristics of studies included for the Systematic review and meta-analysis

Author/s and year of publication	Region	Study Setting	Sample size	Number of Positive	Overall Prevalence	Summary of risk of bias assessment	Reported intestinal parasites	Hygiene factor/s considered for analysis
Abera et al., 2010 [28]	Amhara	Restaurants and cafeterias	384	179	46.6%	Low risk	Al, A, T, Sm, Hn, Gl, H, Ss, and Tt	Hand wash ing after toilet, and food preparation training
Abera et al.,2016 [15]	Amhara	Bahir Dar University cafeterias	410	53	12.93%	Low risk	Al, A, H, Sm, Hn, Gl, Ss and Tt	Medical checkup
Aklilu et al., 2015 [16]	Addis Abeba	Addis Abeba University Cafeterias	172	96	55.81%	Low risk	Al, A, T, Gl, H, and Tt	–
Andargie et al., 2008 [35]	Amhara	Higher institutions Cafeterias in Gondar	127	37	29.10%	Low risk	Al, A, H, Gl, Sm, Ss and Tt	–
Asires et al., 2019 [14]	Amhara	East and West Gojjam Prison	344	123	35.76%	Low risk	Al, A, Hn, Gl, Ev, and H	–
Bedaso, 2010 [23]	Addis Abeba	Orphanage Centers cafeterias	40	14	35%	High risk	Al, A, T, and Gl,	–
Belhu, 2017 [24]	Addis Abeba	Public Hospitals cafeterias	368	119	32.34%	Medium risk	Al, A, T, Gl, H, and Tt	Hand washing before food, hand washing after toilet use, food preparation training, and medical checkup
Dagnew et al., 2012 [18]	Amhara	Gondar University cafeterias	200	50	25%	Low risk	Al, Sm, T, Gl, A, and Ss	Hand washing before food preparation and medical checkup
Desta et al., 2014 [17]	Southern	Hawassa University cafeterias	272	51	18.75%	Medium risk	Al, Sm, T, A, Gl, H and Ss	Hand washing before food preparation
Gebreyesus et al., 2014 [19]	Tigray	Mekelle University cafeterias	307	139	45.27%	Medium risk	Al, Sm, A, T, Hn, Gl, H and Ss	Hand washing before food, food preparation training, and medical checkup
Gezehegn et al., 2017 [25]	Tigray	Restaurants and cafeterias	400	57	14.25%	Low risk	Sm, A, Hn, H, and Gl	food preparation training
Girma et al., 2017 [36]	Oromia	Jimma University cafeterias	94	29	30.85%	Low risk	Al, A, T, Hn, Ev, Gl, H, and Tt	food preparation training and medical checkup
Kebede et al., 2019 [20]	Amhara	Wollo University cafeterias	200	28	14%	Low risk	Al, A, T, and Gl,	–
Nigusse & Kumie, 2012 [12]	Tigray	Mekelle University cafeterias	229	107	46.72%	Low risk	Sm, A, T, Hn, and Gl,	medical checkup
Mama and Alemu, 2016 [37]	Southern	Arbaminch University cafeterias	376	123	32.71%	Low risk	Al, A, T, Gl, H, Ss and Tt	–
Marami et al., 2018 [21]	Oromia	Haramaya University Cafeterias	417	102	24.46%	Low risk	Al, A, T, Hn, Gl, and H,	Hand washing before food, hand washing after toilet use, food preparation training, and medical checkup
Sahlemariam and Mekete, 2001 [22]	Oromia	Higher institution cafeterias in Jimma	101	59	58.41%	Low risk	Al, A, T, Gl, H, and Tt	–
Solomon et al., 2018 [27]	Southern	Restaurants and cafeterias	387	159	41.08%	Low risk	Sm, Al, A, T, Hn, Gl, H, Ss and Tt	–
Tefera, & Mebrie, 2014 [26]	Oromia	Restaurants and cafeterias	118	52	44%	Low risk	Al, A, Ev, Gl, H, and Tt	Hand washing before food, hand washing after toilet use
Wadilo et al.,2016 [13]	Southern	Restaurants and cafeterias	288	97	33.68%	Low risk	Sm, Al, A, Hn, Gl, H, T, Tt, and Ss	Hand washing before food, hand washing after toilet use, food preparation training, and medical checkup

*Abbreviations*: *Al Ascaris lumbricoides*, *A Entamoeba histolytica/dispar*, *T* Taenia species, *H* Hookworms, *Gl Giardia lamblia*, *Hn Hymenlopsis nana*, *Ss Strongloides stercolaris*, *Ev Enterobius vermicularis*, *Sm Schistosoma mansoni* and *Tt Trichuris trichiura*

### Meta-analysis

#### Publication bias assessment

**Fig. 2:**
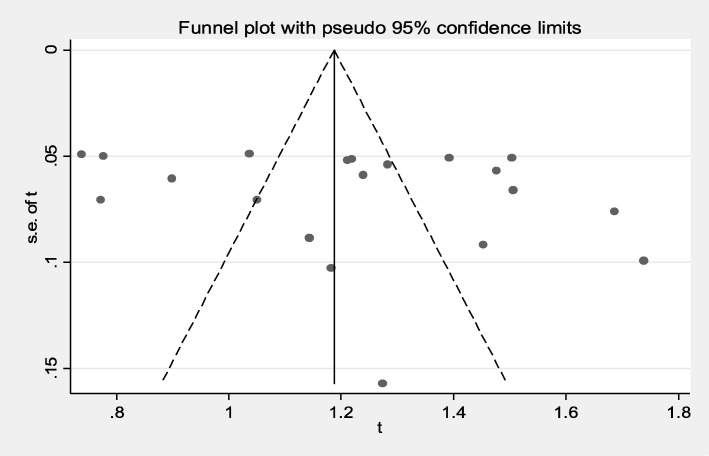
Funnel plot of the arcsine transformed prevalence estimates (t) of intestinal parasites among food handlers at food service establishments in Ethiopia, 2019. Abbreviation: se of t, standard error of t

Symmetrical funnel plot visual inspection (Fig. 2) showed the absence of publication bias which was statistically confirmed by Begg’s test (*P* = 0.314) and Egger’s test (bias coefficient (B) = 4.454 (95%CI = − 4.07–12.98; *P* = 0.287). Hence, we didn’t fill missing theoretical studies by the Duval and Tweedie non-parametric/ trim and fill method.

#### Pooling and heterogeneity analyses

**Fig. 3:**
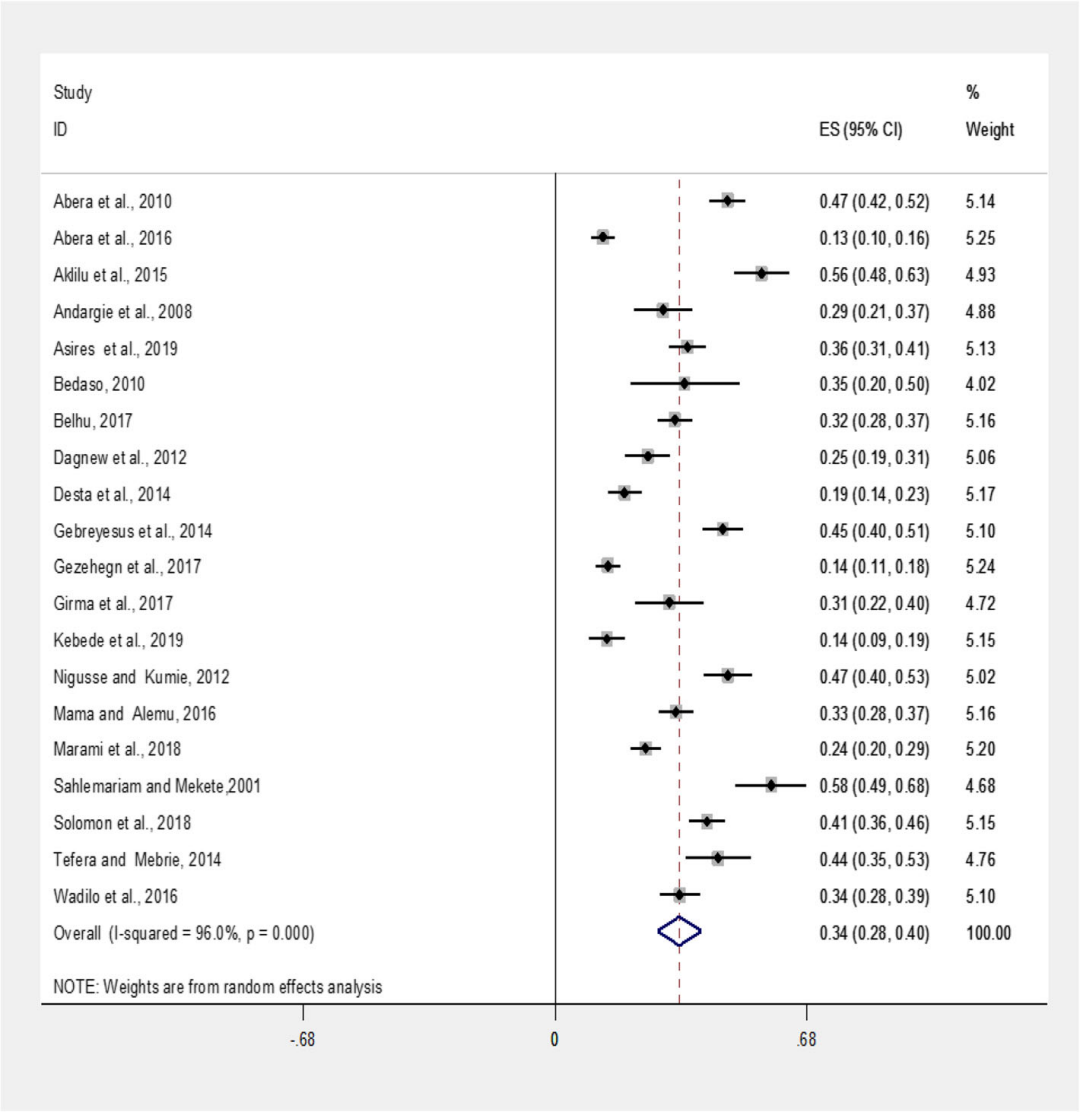
Forest plot depicting pooled prevalence estimate of intestinal parasites among food handlers at food service establishments in Ethiopia, 2019

The prevalence estimates of intestinal parasites among food handlers are presented in a forest plot (Fig. 3). The prevalence estimate varied among studies with considerable heterogeneity (χ2 = 471.80, *P* < 0.001; I^2^ = 96.0%). Thus, we used a random effect model. The pooled prevalence estimate of intestinal parasites among food handlers at food service establishments in Ethiopia which was 33.6% (95%CI: 27.6–39.6%) (Fig. 3). A univariate meta-regression between prevalence and year of publications showed a statistically significant correlation (*P* = 0.033). However, the sample size did not show a statistically significant relationship (*P* = 0.172) (Table 2).

**Table 2:** Univariate meta-regression of factors related to the heterogeneity of intestinal parasites among food handlers at food service establishments in Ethiopia, 2019

Variables	Coefficient	*P*-value
Sample size	−.007682	0.172
Year of publication	−.0324728	0.033

#### Intestinal parasites species specific pooled prevalence

*Entamoeba histolytica/ dispar* pooled prevalence estimate was 11%(95%CI: 7.9–14%) followed by *Ascaris lumbricoides* 8.8%(95%CI: 6.4–11.2%), *Giardia lamblia* 4.5%(95%CI: 3.4–5.6%), Hookworms 2.9%(95%CI: 1.9–4.0%), Taenia species 2.3% (95%CI: 1.4–3.1%), *Strongloides stercolaris* 2.1%(95%CI: 1.1–3%), *Hymenlopsis nana* 2%(95%CI: 1.1–2.9%), *Enterobius vermicularis* 2%(95%CI: 0.8–3.1%), *Trichuris trichiura* 1.3%(95%CI: 0.5–2%) and *Schistosoma mansoni* 1%(95%CI: 0.5–1.5%). *Entamoeba histolytica*/ *dispar* and *Ascaris lumbricoides* were the most dominant intestinal parasites (Table 3).

**Table 3:** Specific intestinal parasites pooled prevalence among food handlers at food service establishments in Ethiopia, 2019

Parasite	No. of studies	Sample size	Positive	Pooled prevalence (95%CI)	I^2^(%)	Heterogeneity	
						Q	P
*Ascaris lumbricoides*	18	4605	388	8.8(6.4–11.2%)	94.1	287.98	< 0.001
*Entamoeba histolytica/ dispar*	20	5234	597	11(7.9–14%)	96	565.79	< 0.001
Taenia species	15	3835	108	2.3(1.4–3.1%)	75.6	57.35	< 0.001
Hookworms	15	4438	155	2.9(1.9–4%)	86.5	103.75	< 0.001
*Giardia lamblia*	20	5234	262	4.5(3.4–5.6%)	78.6	88.73	< 0.001
*Hymenlopsis nana*	10	3260	79	2(1.1–2.9)	79.9	44.80	< 0.001
*Strongloides stercolaris*	9	2751	67	2.1(1.1–3%)	76.2	33.55	< 0.001
*Trichuris trichiura*	11	2825	51	1.3(0.5–2%)	71.1	34.55	< 0.001
*Enterobius vermicularis*	3	556	12	2(0.8–3.1%)	0%	1.08	> 0.582
*Schistosoma mansoni*	10	2944	40	1(0.5–1.5%)	52.3	18.87	< 0.001

#### Sub-group analysis based on geographical regions

**Fig. 4:**
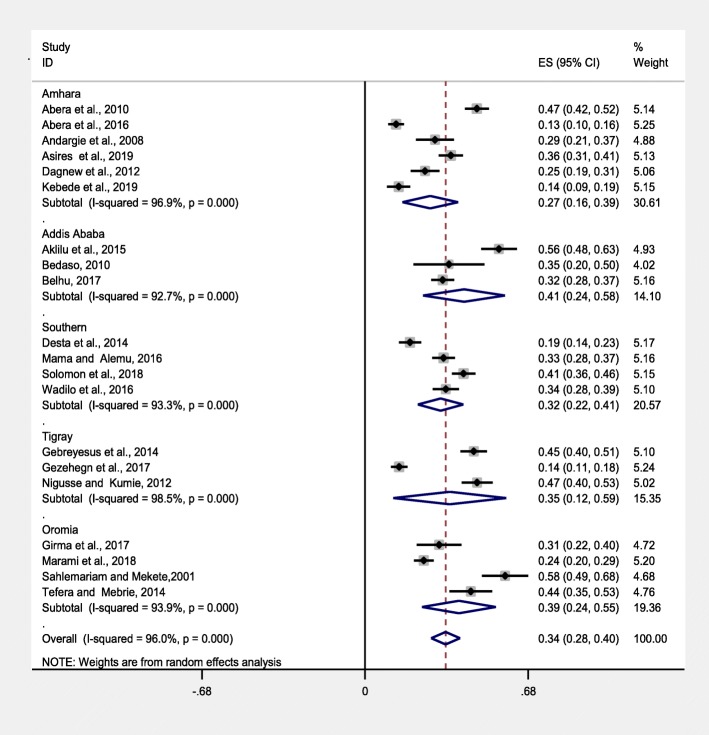
Sub-group pooled prevalence estimate of intestinal parasites in food handlers of food service establishments in Ethiopia, 2019

Furthermore, in this study, we performed a subgroup analysis stratified by geographical regions of the included studies. Accordingly, the highest and the lowest prevalence of intestinal parasites was found in Addis Ababa, 41%(95%CI: 24–58%) and Amhara, 27%(95%CI: 16–39%) regions, respectively (Fig. 4).

#### Hygienic predictors of intestinal parasites among food handlers

**Fig. 5:**
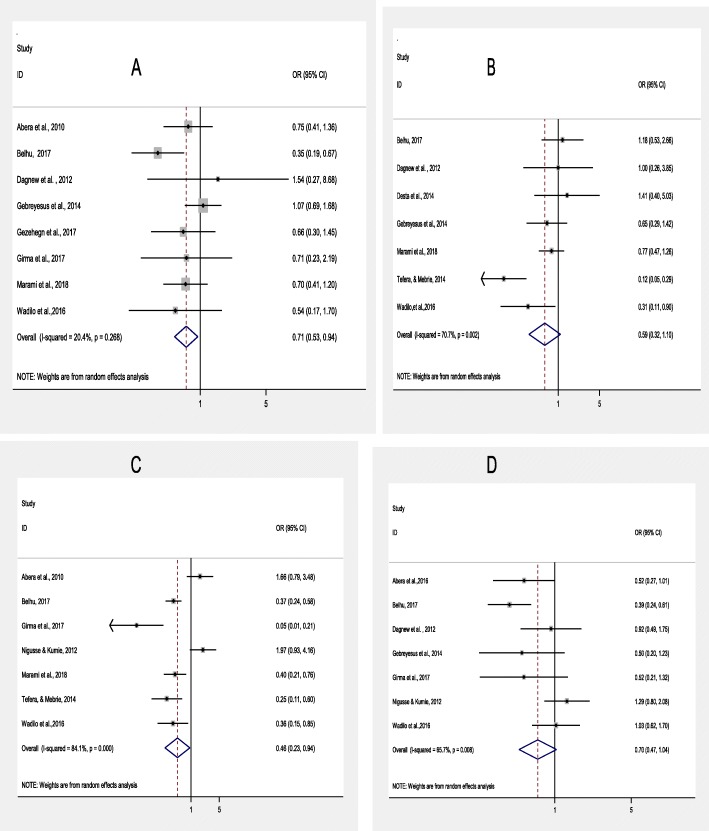
Forest plot showing pooled odds ratio (log scale) of correlation between hygienic practices and intestinal parasitic infections among food handlers at food service establishments in Ethiopia, 2019 (**a**: Food preparation training, **b**: hand washing before food preparation, **c**: washing hands after toilet use, **d**; Medical cheek up)

Food handlers who had food preparation training and medical checkup had 29 and 30% protective effect than their counterparts; for food preparation training, (OR, 0.71; 95% CI: 0.53–.94) and for medical cheek up (OR, 0.70, 95%CI: 0.47–1.04), respectively. Also, food handlers who washed their hands before food preparation had 41% (OR, 0.59; 95%CI: 0.32–1.10) protective effect from gastrointestinal parasites compared to food handlers who had not washed their hands before preparing food. Food handlers who washed their hands after use of toilet use had 54% (OR, 0.46, 95% CI: 0.23–0.94) protective effect against intestinal parasites than those who did not wash their hands after toilet use (Fig. 5 a, b, c, and d).

## Discussion

Healthy people infected with intestinal parasites could be a possible threat to the spread of various pathogens including intestinal parasites for the public in the community. Having better understandings of pooled country-level prevalence estimate of intestinal parasites, endemic species of intestinal parasites, widespread species of intestinal parasites and associated hygienic practice play a crucial role in designing targeted and cost-effective appropriate control mechanisms. Thus, the current systematic review and meta-analysis are the first of its kind in Ethiopia that provides comprehensive helpful information regarding intestinal parasitic infections among food handlers in food service establishments in Ethiopia.

The prevalence of intestinal parasites among food handlers in Ethiopia showed heterogeneity (from 13 to 58%), as the prevalence of intestinal parasite in a community varies depending on socio-demographic factors like sex, age, education status and monthly income and population density and geographic regions such as temperature, humidity/moisture and soil moisture also play a critical role in the transmission of intestinal parasites. Additionally, factors related to hygiene: hand washing practice after the use of toilet, hand washing after touching body parts, hand washing after blowing nose and handwashing before food preparation and handling food, trimming of finger nails, availability of clean water, defecation habit and use of footwear may define the condition of intestine parasite transmission.

The overall pooled prevalence of being infected with at least one intestinal parasite among food handlers of food service establishments in Ethiopia was found to be 33.6% (95%CI: 27.6–39.6%). Though there are no available systematic reviews and meta-analysis conducted on related topics in Ethiopia and elsewhere, the pooled prevalence of intestinal parasitic infections among food handlers in the present study is higher than the primary study conducted in Thailand, (10.3%) [38]. However, the pooled prevalence estimate of intestinal parasites among food handlers in the present study is lower than the primary studies conducted in Venezuela (48.7%) [39], Brazil (47.1%) [40] and Jordan (48.0%) [41]. The relatively lower prevalence of intestinal parasites in this study could be linked to the fact that the present study is a systematic review and meta-analysis which combined twenty primary studies to estimate pooled prevalence. In addition, relative improvements made in safe water supply, health promotion practice, personal and environmental hygiene practice may have contributed in reducing the burden of intestinal parasites in Ethiopia.

In this study, the pooled prevalence of intestinal parasites varies from 1 to 11%. This could be because of the transmission of parasites is influenced by a variety of factors like social, cultural, economic factors and life cycle of parasites. For the spread and rate of intestinal parasitic infections in any area, route of transmission is crucial among others. Intestinal parasites with simple route of transmission, high proliferation capacity and ability to produce cyst or environmental resistance stage could have high prevalence.

Intestinal parasites noticed from food handlers were *Entamoeba histolytica*/ *dispar*, *Ascaris lumbricoides*, *Giardia lamblia*, Hookworms, *Strongloides stercolaris*, *Hymenlopsis nana*, Taenia species, *Enterobius vermicularis*, *Schistosoma mansoni,* and *Trichuris trichiura*. *Entamoeba histolytica*/ *dispar* was the most dominant intestinal parasites among food handlers (11%) while *Schistosoma mansoni* (1%) was the least prevalent. The pooled prevalence of amoebiasis among food handlers in this study is in congruence with the ranges of the nationwide prevalence of amoebiasis in the general population of Ethiopia [42]. This similar prevalence of amoebiasis between food handlers and the general population may be explained by comparable low personal hygiene and environmental sanitation. The highest prevalence of *Entamoeba histolytica*/ *dispar* in the current study may be attributed to widespread open field defecation, the resistance nature of cyst to chlorination, the survival of the cyst for several weeks in the environment and the cyst do not need development and maturation in the environment [43]. Also, most infected individuals are asymptomatic carriers, and they continue to shed eggs for a prolonged time [21].

*Ascaris lumbricoides* was found to be the second most dominant intestinal parasites among food handlers (8.8%). The pooled prevalence estimate of *Ascaris lumbricoides* in the present study is lower than the national prevalence rate of 37% [44] and sub-Sahara Africa prevalence rate of 25% [45]. The differences in the study population may be explained by the inconsistency in the study season, sample size, study methodology, and sensitivity and specificity of the diagnostic technique used. However, the second most prevalence of ascariasis in the present study may be reasoned by a low level of personal hygiene and environmental sanitation, the resistance of *Ascaris* eggs under extreme environmental conditions [46]. Also, gigantic numbers of egg production by female adult worm [47] and thick sticky shell of *Ascaris* eggs facilitate the attachment of *Ascaris* on the surface of human hands, fruits and vegetables [48].

This study has also assessed the correlation between personal hygienic practices and intestinal parasitic infections among food handlers. In the present meta-analysis, having food preparation training and washing hands after toilet use had statistically significant protective effects of food handlers from intestinal parasitic infections by 29, and 54%, respectively. These relatively lower protective effects of hygiene practices from intestinal parasitic infections may be attributed to the presence of confounding factors like poor socioeconomic status, low level of sanitary conditions, safe water supply, latrine utilization and inconsistent adherence to hygienic practice. According to different studies, the transmission of intestinal parasites is affected by access to clean water, socio-economic factors, educational status, individual and public hygienic practice, environmental factors and environmental stages of the parasite [49–51]. This requires the implementation of a holistic approach for the prevention and control of intestinal parasites [52].

This study is not devoid of limitations. Some of the limitations are: 1) multiple intestinal parasitic infections were not given attention by many study reports so that their burden is not reported in the present study. 2) Unevenly distribution of studies to different geographical regions made it impossible to include all regions in sub-group analysis. 3) All studies included in this meta-analysis were cross sectionals, which may be affected by confounding variables. 4) Studies reported only in English were included in this systematic review and meta-analysis.

To the best of the authors’ knowledge, this is the first systematic review with a meta-analysis that provides pooled prevalence estimate of intestinal parasites and associated pooled odds ratio of hygienic factors among food handlers of food service establishments in Ethiopia, and it highlighted implications for practices and further future studies. Some recommendations for further bringing down the magnitude of an intestinal parasite below the level of public health and subsequent future studies are suggested below: 1) pre-placement and periodic screening of food handlers for intestinal parasites should be imposed by employers, managers or owners of food and drink establishments. 2) Food handlers should be trained and educated on regular intervals regarding hygienic practices and modes of transmissions of intestinal parasitic infections. 3) Health institutions, policymakers, and implementers should develop food hygiene training manuals and guidelines to monitor and supervise hygienic practices of food handlers. 4) We also suggest that large scale studies using different standard methods among food handlers of food services establishments, particularly in regions such as Benishangul, Gambela, Afar, and Somali where studies of the prevalence of intestinal parasites among food handlers of food service establishments have not yet been reported.

## Conclusions

In conclusion, the prevalence of intestinal parasites among food handlers of food service establishments in Ethiopia was found to be substantial (33.6%), despite prevalence estimates varied across different geographical regions of the country. Food handlers who washed hands after toilet use and food handlers who had food preparation training had 54 and 29% protection from intestinal parasites as compared to those who did not, respectively. Thus, regular screening, training and personal hygiene practices of food handlers for intestinal parasites should be imposed and monitored by employers, managers or owners of food and drink establishments. Considering varied prevalence among studies and varied protective effects of personal hygienic factors, larger representative studies should be conducted to accurately estimate the magnitude and associated personal hygienic determinants of intestinal parasites among food handlers of food service establishments.

## Supplementary information

**Additional file 1:** **Table S1**. Literature Search strategies for intestinal parasites among food handlers of food service establishments in Ethiopia, April 20, 2019

**Additional file 2: Table S2.** Risk of bias assessment for intestinal parasites among food handlers at food service establishments in Ethiopia, 2019, using Hoy et al., 2012

## Data Availability

All data are presented in the tables shown in the text.

## Abbreviations

B: Bias Coefficient
CI: Confidence Interval
OR: Odds ratio
PRISMA: Preferred Reporting Items for Systematic Reviews and Meta-Analyses
