# Supplementary material for: Intestinal parasites among food handlers of food service establishments in Ethiopia: a systematic review and meta-analysis
Source: BMC Public Health. 2020 Jan 16;20:73. doi: 10.1186/s12889-020-8167-1 (PMC6966842; doi:10.1186/s12889-020-8167-1)
Supplement: Supplementary file 1 — Additional file 1: Table S1. Literature Search strategies for intestinal parasites among food handlers of food service establishments in Ethiopia, April 20, 2019 [file 12889_2020_8167_MOESM1_ESM.docx]

Supplementary file 1: Table S1. Literature Search strategies for intestinal parasites among food handlers of food service establishments in Ethiopia, April 20, 2019

| S.N. | Database | Search strategy | Search results |
| --- | --- | --- | --- |
| 1 | PubMed | (((intestinal parasites OR Parasitic Intestinal Diseases OR intestinal protozoa OR intestinal helminth)) AND Food handlers) AND Ethiopia | 12 |
| 2 | Google scholar | allintitle: Ethiopia intestinal OR parasites OR OR OR Parasitic OR Intestinal OR Diseases OR OR OR intestinal OR protozoa OR OR OR intestinal OR helminth "Food handlers" | 18 |
| 3 | Embase | ('food handlers' OR (('food'/exp OR food) AND handlers)) AND ('ethiopia'/exp OR ethiopia) | 29 |
| 4 | Scopus | TITLE-ABS-KEY ( ''food AND handlers'' AND ethiopia ) | 32 |
| 5 | ovidMEDLINE® | (Food handlers and Ethiopia).mp. [mp=title, abstract, original title, name of substance word, subject heading word, floating sub-heading word, keyword heading word, organism supplementary concept word, protocol supplementary concept word, rare disease supplementary concept word, unique identifier, synonyms] | 24 |
| 6 | Science direct | Find articles with these terms: Food handlers and Ethiopia(Research articles highlighted)= | 90 |
| 7 | Web of sciences | TOPIC: (Food handlers and Ethiopia) | 29 |
| 8 | ProQuest | ab(food handlers) AND ab(Ethiopia) | 43 |
