# Supplementary material for: Intestinal parasites among food handlers of food service establishments in Ethiopia: a systematic review and meta-analysis
Source: BMC Public Health. 2020 Jan 16;20:73. doi: 10.1186/s12889-020-8167-1 (PMC6966842; doi:10.1186/s12889-020-8167-1)
Supplement: Supplementary file 2 — Additional file 2: Table S2. Risk of bias assessment for intestinal parasites among food handlers at food service establishments in Ethiopia, 2019, using Hoy et al., 2012 [file 12889_2020_8167_MOESM2_ESM.docx]

| Study ID | Representation | Sampling | Random selection | Non response bias | Data collection | Case Definition | Reliability and validity of study tool | Method of data collection | Prevalence period | Numerator and denominator | Overall  Assessment |
| --- | --- | --- | --- | --- | --- | --- | --- | --- | --- | --- | --- |
| [1] | low risk | low risk | low risk | Uncertain | low risk | low risk | low risk | Medium risk | Low risk | Medium risk | Low risk |
| [2] | Low risk | Low risk | Low risk | Uncertain | Low risk | Low risk | Low risk | Low risk | Low risk | Low risk | Low risk |
| [3] | Low risk | Low risk | Low risk | Low risk | Low risk | Low risk | Low risk | Low risk | Low risk | Low risk | Low risk |
| [4] | Medium risk | Low risk | Low risk | Low risk | Low risk | Low risk | Low risk | Low risk | Low risk | Low risk | Low risk |
| [5] | Low risk | Low risk | Low risk | Low risk | Low risk | Low risk | Low risk | Low risk | Low risk | Low risk | Low risk |
| [6] | High risk | High risk | uncertain | Uncertain | High risk | Low risk | High risk | Low risk | Low risk | Low risk | High risk |
| [7] | Medium risk | High risk | Medium risk | Low risk | Low risk | Low risk | Low risk | Low risk | Low risk | Low risk | Medium risk |
| [8] | Low risk | Low risk | Low risk | Uncertain | Low risk | Low risk | Low risk | Low risk | Low risk | Low risk | Low risk |
| [9] | Low risk | uncertain | uncertain | uncertain | Low risk | Low risk | Low risk | Low risk | Low risk | Low risk | Medium risk |
| [10] | Low risk | uncertain | uncertain | uncertain | Low risk | Low risk | Low risk | Low risk | Low risk | Low risk | Medium risk |
| [11] | Low risk | Low risk | Low risk | Low risk | Low risk | Low risk | Low risk | Low risk | Low risk | Low risk | Low risk |
| [12] | Low risk | Low risk | Low risk | Low risk | Low risk | Low risk | Low risk | Low risk | Low risk | Low risk | Low risk |
| [13] | Low risk | Low risk | Low risk | Uncertain | Low risk | Low risk | Low risk | Low risk | Low risk | Low risk | Low risk |
| [14] | Low risk | Low risk | Low risk | Low risk | Low risk | Low risk | Low risk | Low risk | Low risk | Low risk | Low risk |
| [15] | Low risk | Low risk | Low risk | Low risk | Low risk | Low risk | Low risk | Low risk | Low risk | Low risk | Low risk |
| [16] | Low risk | Low risk | Not clear | Not clear | Low risk | Low risk | Low risk | Low risk | Low risk | Low risk | low risk |
| [17] | Low risk | Not clear | Uncertain | Low risk | Low risk | Low risk | Low risk | Low risk | Low risk | Low risk | Low risk |
| [18] | Low risk | Low risk | Low risk | Low risk | Low risk | Low risk | Low risk | Low risk | Low risk | Low risk | Low risk |
| [19] | Low risk | Low risk | Low risk | Low risk | Low risk | Low risk | Low risk | Low risk | Low risk | Low risk | Low risk |
| [20] | Low risk | Low risk | Low risk | Uncertain | Low risk | Low risk | Low risk | Low risk | Low risk | Low risk | Low risk |

1. Representation: Was the study population a close representation of the national population?

2. Sampling: Was the sampling frame a true or close representation of the target population?

3. Random selection: Was some form of random selection used to select the sample OR was a census undertaken?

4. Non-response bias: Was the likelihood of non-response bias minimal?

5. Data collection: Were data collected directly from the subjects?

6. Case definition: Was an acceptable case definition used in the study?

7. Reliability and validity of study tool: Was the study instrument that measured the parameter of interest show to have reliability and validity?

8. Data collection: Was the same mode of data collection used for all subjects?

9. Prevalence period: Was the length of the prevalence period for the parameter of interest appropriate?

10. Numerators and denominators: Were the numerator(s) and denominator(s) for the parameter of interest appropriate?

The overall risk of bias was then rated based on the number of high risk of bias per study: low (≤2), moderate (3–4), and high (≥5).

1. Abera B, Biadegelgen F, Bezabih B: **Prevalence of Salmonella typhi and intestinal parasites among food handlers in Bahir Dar Town, Northwest Ethiopia**. *Ethiopian Journal of Health Development* 2010, **24**(1).

2. Abera B, Yitayew G, Amare H: **Salmonella serotypeTyphi, Shigella, and intestinal parasites among food handlers at Bahir Dar University, Ethiopia**. *Journal of Infection in Developing Countries* 2016, **10**(2):121-126.

3. Aklilu A, Kahase D, Dessalegn M, Tarekegn N, Gebremichael S, Zenebe S, Desta K, Mulugeta G, Mamuye Y, Mama M: **Prevalence of intestinal parasites, salmonella and shigella among apparently health food handlers of Addis Ababa University student's cafeteria, Addis Ababa, Ethiopia**. *BMC Research Notes* 2015, **8**(1).

4. Andargie G, Kassu A, Moges F, Tiruneh M, Huruy K: **Prevalence of bacteria and intestinal parasites among food-handlers in Gondar town, northwest Ethiopia**. *Journal of health, population, and nutrition* 2008, **26**(4):451.

5. Asires A, Wubie M, Reta A: **Prevalence and Associated Factors of Intestinal Parasitic Infections among Food Handlers at Prison, East and West Gojjam, Ethiopia**. *Advances in medicine* 2019, **2019**.

6. Bedaso S: **Prevalence of intestinal parasitic infections among food handlers and microbial safety of ready-to-eat foods in selected orphanage centers in Addis Ababa**. *MSc Thesis.* Addis Ababa University; 2010.

7. Belhu T: **Prevalence of Intestinal Parasites and Gastrointestinal Carriage of Gram Negative Enteric Bacteria among Apparently Healthy Food Handlers of Public Hospitals, Addis Ababa, Ethiopia**. *MSc Thesis.* Addis Ababa University; 2017.

8. Dagnew M, Tiruneh M, Moges F, Tekeste Z: **Survey of nasal carriage of Staphylococcus aureus and intestinal parasites among food handlers working at Gondar University, Northwest Ethiopia**. *BMC public health* 2012, **12**(1):837.

9. Desta M, Asrat D, Woldeamanuel Y, Nigusie D: **Prevalence of intestinal parasites and Salmonella and Shigella among food handlers at food service establishments in the main campus and Health Sciences College of Hawassa University, Hawassa, Ethiopia**. *The Ethiopian Journal of Health Development* 2014, **28**(1).

10. Gebreyesus A, Adane K, Negash L, Asmelash T, Belay S, Alemu M, Saravanan M: **Prevalence of Salmonella typhi and intestinal parasites among food handlers in Mekelle University student cafeteria, Mekelle, Ethiopia**. *Food Control* 2014, **44**:45-48.

11. Gezehegn D, Abay M, Tetemke D, Zelalem H, Teklay H, Baraki Z, Medhin G: **Prevalence and factors associated with intestinal parasites among food handlers of food and drinking establishments in Aksum Town, Northern Ethiopia**. *BMC public health* 2017, **17**(1):819.

12. Girma H, Beyene G, Mekonnen Z: **Prevalence of intestinal parasites among food handlers at cafeteria of Jimma University Specialized Hospital, Southwest Ethiopia**. *Asian Pacific Journal of Tropical Disease* 2017, **7**(8):467-471.

13. Kebede E, Seid A, Akele S: **Prevalence and associated risk factors of intestinal parasitic infections among asymptomatic food handlers in Wollo University student’s cafeteria, Northeastern Ethiopia**. *BMC research notes* 2019, **12**(1):139.

14. Nigusse D, Kumie A: **Food hygiene practices and prevalence of intestinal parasites among food handlers working in Mekelle university student’s cafeteria, Mekelle**. *Garjss* 2012, **1**(4):65-71.

15. Mama M, Alemu G: **Prevalence and factors associated with intestinal parasitic infections among food handlers of Southern Ethiopia: Cross sectional study Infectious Disease epidemiology**. *BMC Public Health* 2016, **16**(1).

16. Marami D, Hailu K, Tolera M: **Prevalence and associated factors of intestinal parasitic infections among asymptomatic food handlers working at Haramaya University cafeterias, eastern Ethiopia**. *Annals of occupational and environmental medicine* 2018, **30**:53.

17. Sahlemariam Z, Mekete G: **Examination of fingernail contents and stool for ova, cyst and larva of intestinal parasites from food handlers working in student cafeterias in three Higher Institutions in Jimma**. *Ethiopian Journal of Health Sciences* 2001, **11**(2).

18. Solomon FB, Wada FW, Anjulo AA, Koyra HC, Tufa EG: **Burden of intestinal pathogens and associated factors among asymptomatic food handlers in South Ethiopia: emphasis on salmonellosis**. *BMC research notes* 2018, **11**(1):502.

19. Tefera T, Mebrie G: **Prevalence and predictors of intestinal parasites among food handlers in Yebu town, southwest Ethiopia**. *PLoS One* 2014, **9**(10):e110621.

20. Wadilo F, Solomon F, Arota A, Abraham Y: **Intestinal parasitic infection and associated factors among food handlers in South Ethiopia: a case of Wolaita Sodo Town**. *Journal of Pharmacy and Alternative Medicine* 2016, **12**:5-10.
